# An advertising poster of “Aama” a poor french acromegalic giantess sold twice!

**DOI:** 10.1007/s40618-026-03001-3

**Published:** 2026-07-22

**Authors:** Wouter W. de Herder

**Affiliations:** Department of Internal Medicine, Sector of Endocrinology, Rg520, Dr. Molewaterplein 40, 3015 GD Rotterdam, The Netherlands

An advertising poster created around 1890 by Gérard Marci (1852–1928), who ran a well-known printing business named “Imprimerie Marci” in Brussels, Belgium, showing from left to right: Pierre Clément, Aline Bataillard (“Aama”) and Joséphine Guilhe (“Princess Josepha”) (Fig. 1). The poster exists in two known versions and was used to advertise for public appearances of “Aama”. The artist who drew it remains unknown.

Aline Bataillard (“Aama”) was a 203 cm (6 feet 8 inches) tall acromegalic giantess who was born on March 5, 1872 in Le Bouchaud in Jura, France and who died on February 27, 1893 in Des Moines in Iowa, USA.

The biography of Aline Bataillard is one of the most heartbreaking stories on the lives of circus, fair, or sideshow giants [1].

Aline Bataillard was the 15th child of Auguste Bataillard, farmer, woodcutter, ironworker, and day labourer and Anna Appoline Landry. When she was 12 years old, Aline was already more than 183 cm (6 feet) tall. Although her final height was 203 cm (6 feet 8 inches), her billed height for commercial purposes was more than 244 cm (8 feet) (Fig. 1). She wore European size 50 / US size 17 shoes and was advertised weighing 130.5 kg (288 pounds).

Flyers and advertisements reported that Aline also drank “20 to 25 quarts” (19–23 L) per day and “eats about six times as much as any ordinary person”. This might indicate that she had developed diabetes mellitus. However, her poor parents could not afford the enormous amounts of food and drinks Aline Bataillard needed.

At the age of 17, Aline Bataillard visited the Pentecost fair in Dole, Jura, France, with her grandfather who sold her for 700 francs to the professional showmen Pierre Clément and his son Auguste Clément. With these two showmen she would run between June 1889 and October 1892 an exhausting tour across France (Marseille, Toulouse, Pau, Bordeaux, Amiens, Saint-Quentin, Troyes and Paris), Mainland Europe (Brussels and Berlin) and Great Britain (London, Cardiff and Liverpool).

In September 1890 at the fair in Toulouse, France, Aline Bataillard was not any longer presented as Aline, but as Marie-Emma. Pierre and August Clément used the birth certificate of Aline’s four years younger sister, Marie Emma Bataillard, who was born on 16 August 1876. Herewith the showmen committed for the first time passport and identity fraud.

In November 1890 at the “Foire Saint-Martin” (“St. Martin’s Fair”) in Pau, France, Aline (Marie Emma) was being advertised as 14 years old and she was in the company of a 14-year-old 70 cm (2 feet 3.6 inches) tall “lilliputian”. In reality, this midget lady was the 6-year-old Joséphine Guilhe, who was of normal health. She was used for contrasting the height differences between herself and the giantess (Fig. 2).

Late 1892, a US tour was planned. Aline Bataillard who was already very exhausted, weak and needed a rod to support herself arrived at Ellis Island, New York, USA on 14 November 1892. She was in the company of the then 48-year-old Peter Clement (Pierre Clément), listed on the US immigration form as “showman”, his 26-year-old daughter Bertha Clement ((Berthe Clément, listed as “Matron”), his 21-year-old son Augustus “Gus” Clement (Auguste Clément), also listed as “showman” and the 8-year-old Joséphine Guilhe (listed as “spinster”!) and presented as Berthe’s daughter. Aline Bataillard was also listed as a 16-year-old “Spinster.” On stage, Joséphine was to be presented as a twenty-one-year-old lilliputian / dwarf named “Princess Josepha”, Aline’s older sister to make the height contrasting picture more complete. Aline Bataillard’s (Marie-Emma Bataillard’s) on-stage aliases were “Emma Alma”, “Emma Aline”, “Aama”, “Ama”, “Lady Aama”, and “Emma” (Fig. 2).

Between 21 November 1892 and 27 February 1893, the group was showing at New-York, Chicago, Boston, Pittsburgh, Altoona and finally arrived in Des Moines.

Being completely exhausted, Aline Bataillard died as Marie Emma Bataillard after a violent coughing fit on 27 February 1893 in Des Moines, Iowa, USA. The cause of death was reported as collapse after a common cold. In reality, Marie Emma Bataillard passed away on 19 October 1955 in Montferrand-le-Château, France.

For the second time Aline Bataillard was sold: Pierre Clément sold her body for $200 to the University of Iowa at Iowa City. After arrival in Iowa City on 4 march 1893., the corpse of Aline Bataillard was examined and measured by Dr. Woods Hutchinson (1862–1930), lecturer on anatomy at the State University of Iowa in Iowa City and he subsequently supervised the dissection of the body of the giantess.

Aline Bataillard’s corpse was found to be 6 feet 7.75 inches (203 cm.) long. Her true height while alive would exceed this slightly, according to Woods Hutchinson. The foot length was 13¾ inches (35 cm.). Her body was severely emaciated. There were typical acromegalic features (like: large hands & prognathism), the phenotype was female (an uterus and streak ovaries were found), but there was complete atrophy of the mammary glands and clitoromegaly (during lifetime, it was suggested that Aline Bataillard was a hermaphrodite). The sella turcica was enlarged 1¼ x 1½ inch (3.2 × 3.8 cm) and the pituitary gland was the size of a pigeon’s egg. Severe generalized osteoporosis was found.

Woods Hutchinson published Aline Bataillard’s case in 1895 [2]. He concluded that Aline Bataillard had suffered from acromegaly and he asked the question: “would it not appear probable that at least one form of giantism is merely acromegaly beginning in fœtal or infant life?” In 1895, the general opinion in the medical field was that acromegaly and giantism were two separate disorders, but Woods Hutchinson at least made an attempt to link these two disorders.

The bones of Aline Bataillard were kept as museum specimens in the Anatomical Department of the University of Iowa at Iowa City but were too fragile, or were falling apart, making it impossible to be mounted into a skeleton. Aline Bataillard’s bones were destroyed when the medical school’s building burned down to the ground on 10 March 1901.

**Fig. 1:**
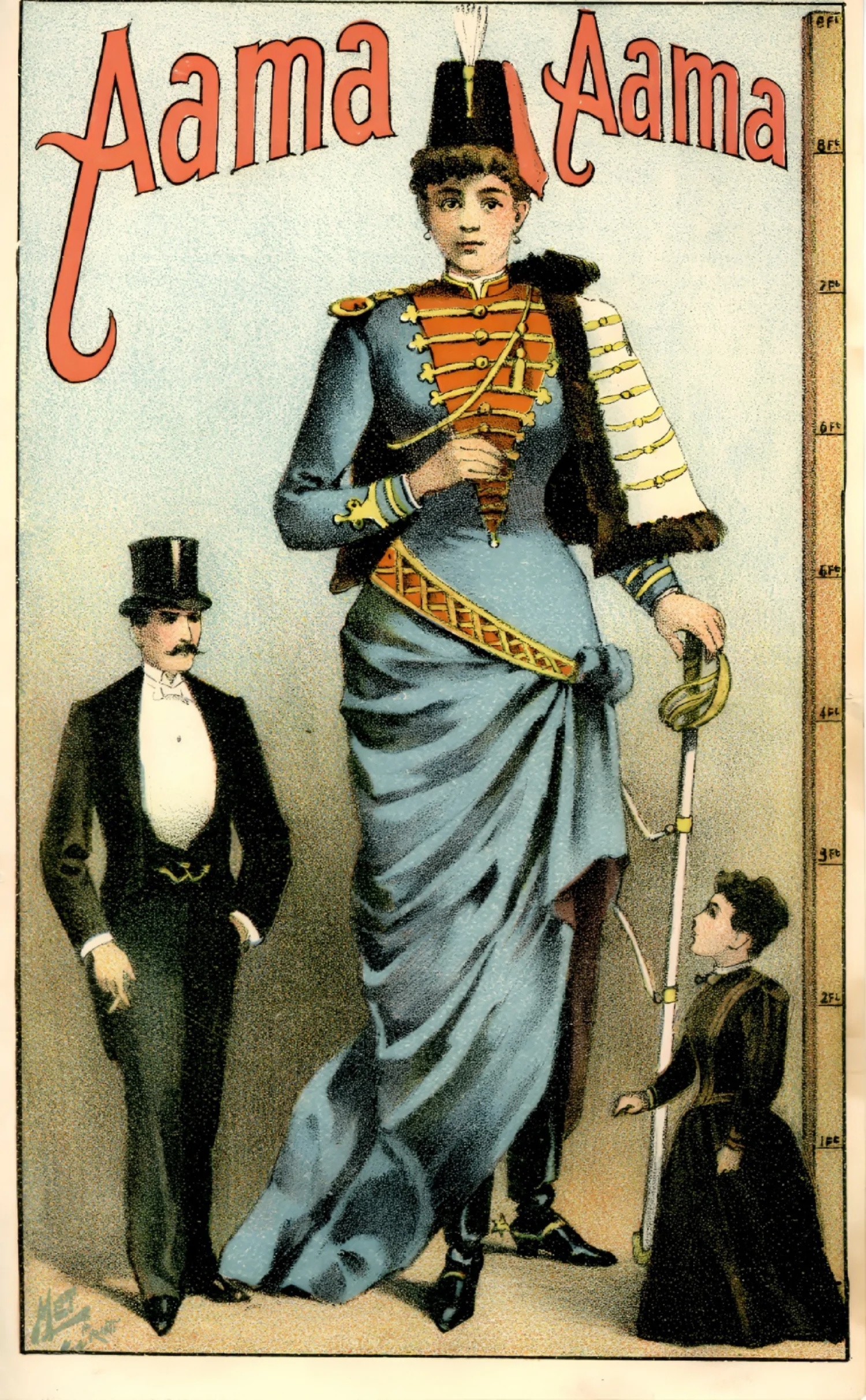
Poster advertising “Aama” the giantess created ca. 1890 by Gérard Marci from “Imprimerie Marci” in Brussels, Belgium, showing from left to right: Pierre Clément, Aline Bataillard (“Aama”) and Joséphine Guilhe. “Aama” was advertised as 14 years old and 244 cm (8 feet) tall. Joséphine Guilhe was advertised as 14 years old and 70 cm (2 feet 3.6 inches) tall. Picture from the collection of Warren Raymond, Silver Spring, MD, USA

**Fig. 2:**
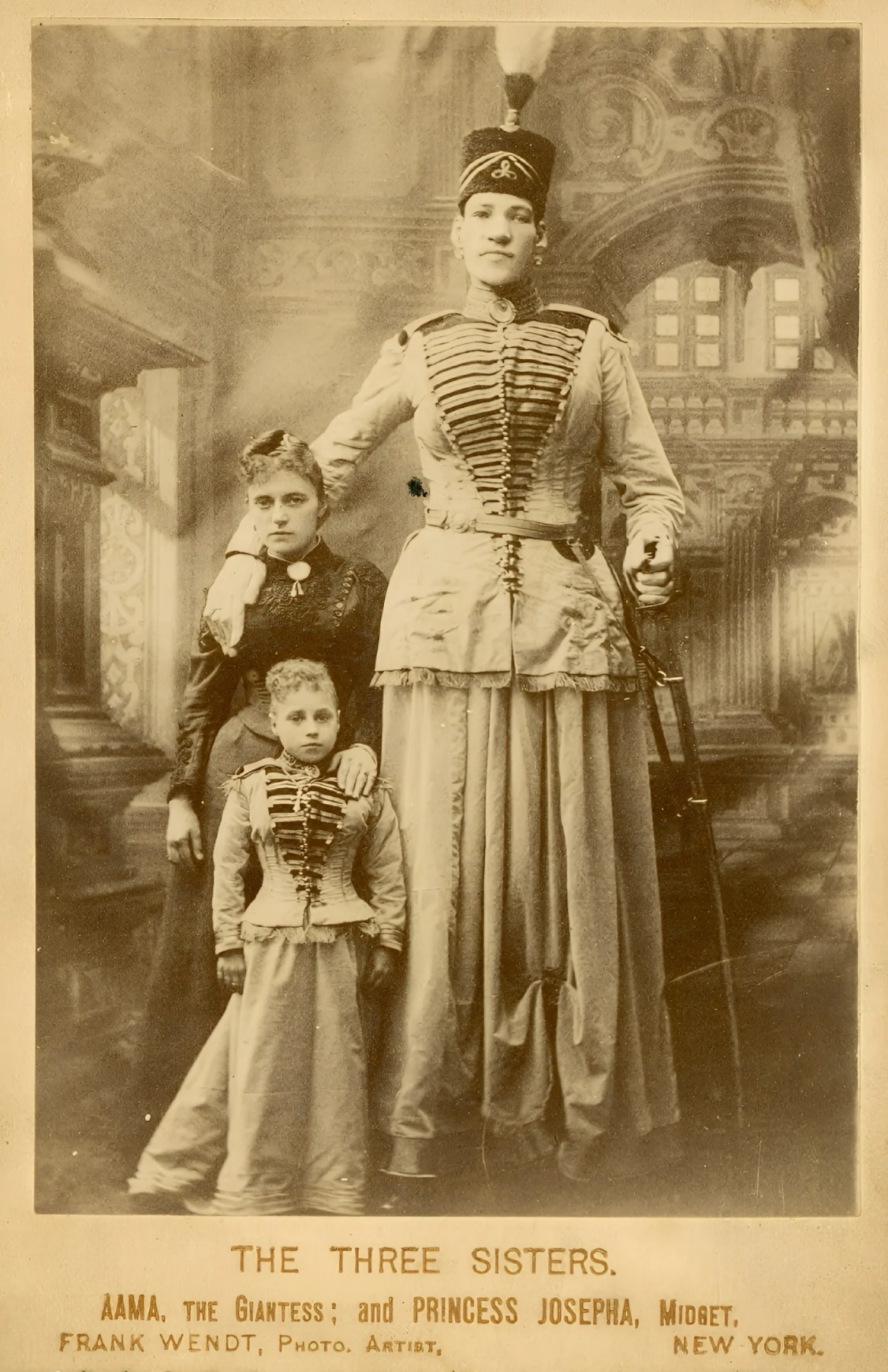
Cabinet card named “The Three Sisters” by photographers Charles Eisenmann (1855–1927) and Frank Wendt (1858–1930) from New York City, New York, USA. The picture most probably was taken in November 1892. It shows on the left in the front “Princess Josepha” a 21-year-old “lilliputian / dwarf” lady of 91.5 cm (3 feet) tall and claimed to be the sister of the giantess. In reality she was the 8-year-old Joséphine Guilhe, who was of normal health. On the left in the back is shown 26-year-old Berthe Clément / Bertha Clement. At the right is shown the giantess “Aama”, 203 cm (5 feet 8 inches) tall Aline Bataillard (1872–1893). The three ladies were not sisters and did not have any family bonds between them. Picture from the collection of Warren Raymond, Silver Spring, MD, USA

## References

[1] Pascale Bonnier La pitoyable faiblesse des géants: Aline Bataillard. Academia.edu 2023. https://www.academia.edu/113163996/La_pitoyable_faiblesse_des_géants_Aline_Bataillard

[2] Woods Hutchinson A case of acromegaly in a giantess. Am J Med Sci 1895; 110: 190–201

